# Patient-specific virtual three-dimensional surgical navigation for gastric cancer surgery: A prospective study for preoperative planning and intraoperative guidance

**DOI:** 10.3389/fonc.2023.1140175

**Published:** 2023-02-21

**Authors:** Sung Hyun Park, Ki-Yoon Kim, Yoo Min Kim, Woo Jin Hyung

**Affiliations:** ^1^ Department of Surgery, Yonsei University College of Medicine, Seoul, Republic of Korea; ^2^ Gastric Cancer Center, Yonsei Cancer Center, Yonsei University Health System, Seoul, Republic of Korea; ^3^ Vision AI, Hutom, Seoul, Republic of Korea

**Keywords:** surgical navigation, gastric cancer, robotic gastrectomy, image-guided surgery, patient-specific 3-D model

## Abstract

**Introduction:**

Abdominal computed tomography (CT) can accurately demonstrate organs and vascular structures around the stomach, and its potential role for image guidance is becoming increasingly established. However, solely using two-dimensional CT images to identify critical anatomical structures is undeniably challenging and not surgeon-friendly. To validate the feasibility of a patient-specific 3-D surgical navigation system for preoperative planning and intraoperative guidance during robotic gastric cancer surgery.

**Materials and methods:**

A prospective single-arm open-label observational study was conducted. Thirty participants underwent robotic distal gastrectomy for gastric cancer using a virtual surgical navigation system that provides patient-specific 3-D anatomical information with a pneumoperitoneum model using preoperative CT-angiography. Turnaround time and the accuracy of detecting vascular anatomy with its variations were measured, and perioperative outcomes were compared with a control group after propensity-score matching during the same study period.

**Results:**

Among 36 registered patients, 6 were excluded from the study. Patient-specific 3-D anatomy reconstruction was successfully implemented without any problems in all 30 patients using preoperative CT. All vessels encountered during gastric cancer surgery were successfully reconstructed, and all vascular origins and variations were identical to operative findings. The operative data and short-term outcomes between the experimental and control group were comparable. The experimental group showed shorter anesthesia time (218.6 min *vs*. 230.3 min; *P*=0.299), operative time (177.1 min *vs*. 193.9 min; *P*=0.137), and console time (129.3 min *vs*. 147.4 min; *P*=0.101) than the control group, although the differences were not statistically significant.

**Conclusions:**

Patient-specific 3-D surgical navigation system for robotic gastrectomy for gastric cancer is clinically feasible and applicable with an acceptable turnaround time. This system enables patient-specific preoperative planning and intraoperative navigation by visualizing all the anatomy required for gastrectomy in 3-D models without any error.

**Clinical trial registration:**

Clinicaltrials.gov, identifier NCT05039333.

## Introduction

Gastrectomy with systemic lymphadenectomy is the most important treatment for gastric cancer (1). Skeletonizing and managing vessels are crucial for thorough lymphadenectomy during gastrectomy since lymph nodes are located along the vessels (2). Thus, anatomical information of organs and vessels and their variations around the stomach is essential for safe and complete lymphadenectomy during radical gastrectomy (3–6).

Recent technological developments enable more specific and easily identifiable virtual modeling of anatomical structures from computed tomography (CT). CT effectively detects relevant and critical variations of the vascular anatomy (7). CT can accurately demonstrate organs and vascular structures around the stomach, and its potential role for image guidance is being increasingly established (3, 8). However, solely using two-dimensional computed tomography images to identify critical structures is undeniably difficult. The interpretation of CT is limited by the ability to discriminate details in CT images, even for expert radiologists. Even after identifying vessels, these images are not surgeon-friendly since this information cannot be easily delivered to surgeons during surgery. Moreover, intraoperative use of image guidance for minimally invasive gastrectomy remains challenging (5, 6, 9).

Although attempts to accurately provide vascular anatomy using 2-D CT images have been continued, identifying the positional relationship between structures is still difficult unless serial images are checked due to the limitations of 2-D images (3). Also, regarding vessels and organs in the abdominal cavity are not fixed structures different from neurosurgery or orthopedics area, developing surgical navigation through CT images in the abdominal cavity was technically challenging. To overcome these challenges, a new virtual surgical navigation system that provides patient-specific 3-D vascular information with a pneumoperitoneum model (RUS™, Hutom, Seoul, Korea) was developed. RUS™ is a software that offers a virtual surgical environment with pneumoperitoneum resembling actual operative environment. This system provides 3-D virtual anatomy and surgical instrumental models that enable preoperative planning, anatomy simulation, and intraoperative anatomy navigation. We aimed to evaluate the feasibility of a patient-specific virtual surgical navigation system for preoperative planning and intraoperative guidance with 3-D vascular imaging during robotic gastrectomy with systemic lymphadenectomy. We also assessed the accuracy of vascular anatomy around the stomach` obtained from the patient-specific surgical navigation system and compared it with surgical findings. Additionally, surgical outcomes were compared between robotic gastrectomy with patient-specific virtual surgical navigation system and without it.

## Materials and methods

### Patients

This study was designed as a prospective single-arm observational study allowing for comparison with the control group after propensity-score matching. We enrolled gastric cancer patients who were scheduled for robotic surgery for distal subtotal gastrectomy. We included patients aged 18 years or older who had an abdominopelvic CT according to the established protocol. We excluded patients whose major vascular structures around the stomach had been altered due to previous surgery and those with history of any gastric surgery. We also excluded patients who could not have a CT scan due to contrast agent allergy, creatinine level above 1.5 times the normal maximum, and claustrophobia. To perform robotic subtotal gastrectomy using surgical navigation in 30 patients, this clinical trial was designed to enroll 36 patients considering 20% drop out and exclusion of enrolled participants during 6 months enrollment period.

The control group was selected among 175 patients who took CT angiography with an established protocol capable of 3-D model reconstruction between September 2014 and September 2021 from the prospectively collected gastric cancer database. We used the same eligibility criteria for the control and the experimental group. After excluding 28 patients who underwent total gastrectomy or proximal gastrectomy, 147 gastric cancer patients underwent robotic distal gastrectomy. Of these 147 patients, we used propensity-score matching for control group selection to balance the two groups for different clinical and surgical features. A control group was selected using 1:1 propensity-score matching with covariates of patient demographics (age, sex, body mass index) and operative factors (extent of lymph node dissection and reconstruction type). We set the caliper value of 0.1 for 1:1 matching using the nearest method without replacement.

This study was approved by the Institutional Review Board (IRB) of Severance Hospital, Yonsei University Health System (1-2021-0036). Written informed consent was obtained from patients after a full explanation of the study. Informed consent for patients included in the control group was waived by the IRB because of its retrospective nature.

### Schema of RUS™, the patient-specific virtual surgical navigation system

**Figure 1:**
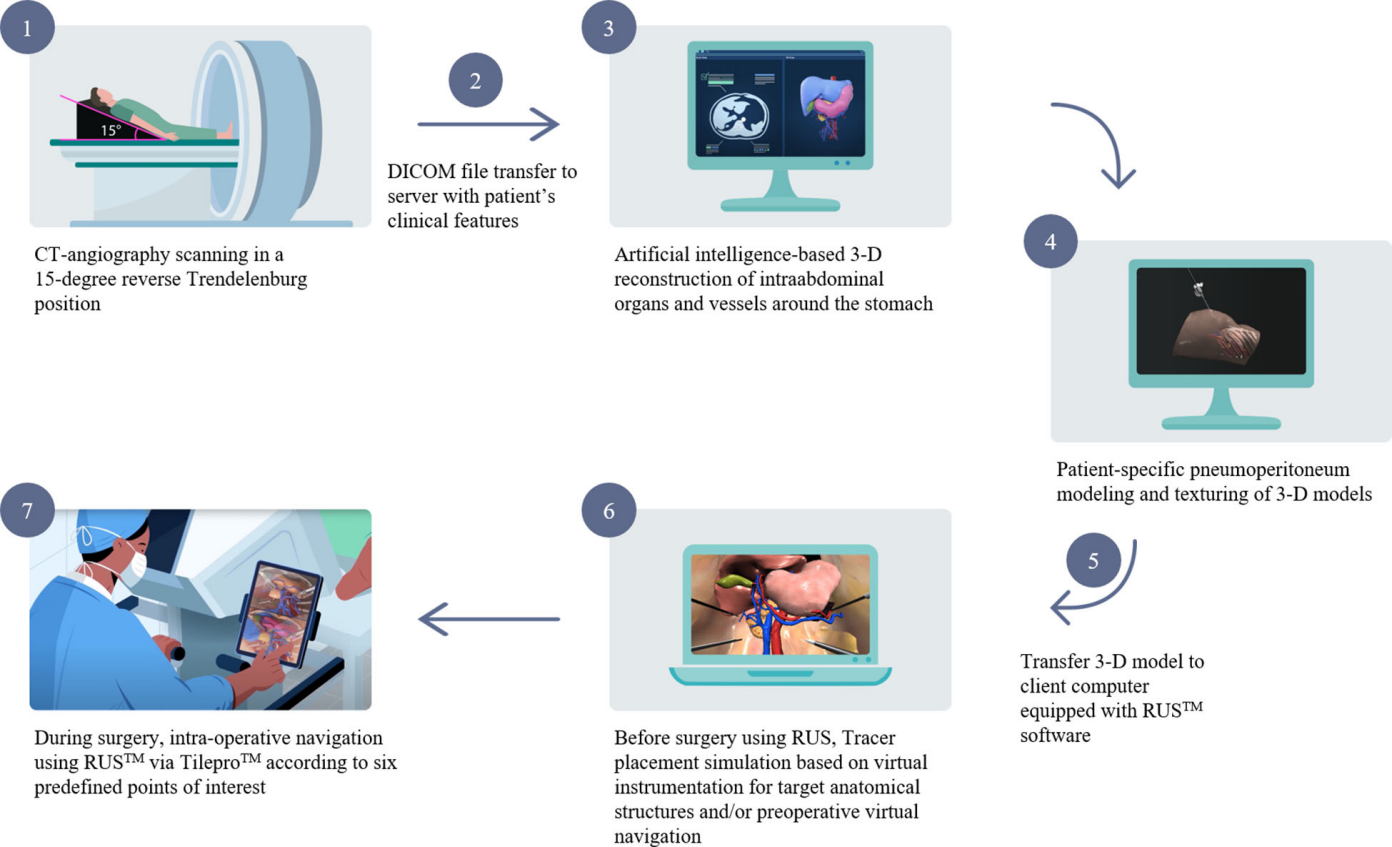
Schema of the patient-specific virtual 3-D surgical navigation system.

**Figure 2:**
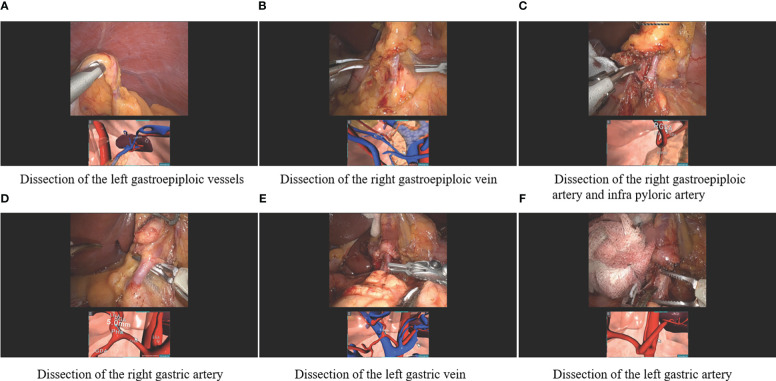
Robotic gastrectomy procedures using the patient-specific virtual 3-D surgical navigation system according to the six pre-defined points of interest **(A)** Dissection of the left gastroepiploic vessels, **(B)** Dissection of the right gastric vein, **(C)** Dissection of the right gastroepiploic artery and infra pyloric artery, **(D)** Dissection of the right gastric artery, **(E)** Dissection of the left gastric vein, and **(F)** Dissection of the left gastric artery.

The schema of RUS™ consists of the following processes: 1) Taking abdominopelvic CT angiography in a 15-degree reverse Trendelenburg position. 2) Transferring the digital imaging and communications in medicine (DICOM) file of the CT angiography and patient’s clinical features to a server. 3) Artificial intelligence-based 3-D reconstruction of intraabdominal organs and vessels around the stomach. 4) Texturing of the 3-D reconstructed intraabdominal organs and vessels and generation of patient-specific pneumoperitoneum model. 5) Transferring 3-D reconstructed models to client computer equipped with RUS™. 6) Virtual trocar placement and instrumentation simulation based on preoperative virtual simulation. 7) Intraoperative anatomy navigation using RUS™ according to six pre-defined points of interest (**Figure 1**). The six pre-defined points of interest included in the RUS™ consist of left gastroepiploic artery and vein area, right gastroepiploic vein area, right gastroepiploic and infra-pyloric artery area, right gastric artery area, left gastric vein area, and left gastric artery area (**Figure 2**).

### Computed tomographic technique

All patients in the experimental group underwent CT scanning with multidetector row CT (Revolution EVO; GE Medical Systems, Milwaukee, WI, USA). Before the CT scan, all patients received an intravenous injection of 10-mg butylscopolamine bromide (Buscopan, Boehringer Ingelheim, Seoul, Korea) in at least 4-hour fast state to minimize bowel peristalsis and induce hypotonia. One pack (4 grams) of sodium bicarbonate/tartaric acid (Bargin effervescent granule, With Health Care, Seoul, Korea) with a minimal amount of water (<10 mL) was administered orally 3 minutes before the CT scan to establish gastric distention without positive oral contrast. To develop a surgery-oriented 3-D model, CT scan was performed in a reverse Trendelenburg position. All patients were placed in a 15-degree reverse Trendelenburg position at the CT table using a wedge on the back of the patients to minimize the difference in positional deformation of internal organs caused by the difference between the supine position and the 15-degree reverse Trendelenburg surgical position. Scans were started 2 minutes after positioning to maximize the positional changes of internal organs by gravity. All patients received 2 mL/kg of an iodinated non-ionic vascular radiocontrast agent, Iohexol (Hexosure 350 inj, Pharvis Korea, Seoul, Korea) intravenously using an automatic power injector with standardized multiphase injection protocol at a rate of 3 mL/second followed by saline flushing. Scans were acquired in a craniocaudal direction during a single breath-hold with the following parameters: detector collimation of 64 rows × 0.625 mm; rotation time, 0.5 seconds; pitch, 0.984; tube voltage, 120 kVp, and tube current, 150-350 mA with tube current modulation.

A bolus-tracking program was used to commence diagnostic CT data acquisition after the intravenous injection of the contrast agent. The region-of-interest cursor for bolus tracking was placed in the descending aorta at the first lumbar vertebra level for real-time serial monitoring. Early arterial and portal phase images were commenced at 6 and 55 seconds, respectively, after the trigger (trigger threshold level: 100 HU). Axial CT images were reconstructed with a 1 mm section thickness and a 1 mm interval for 3D reconstruction, and maximum intensity projection (MIP) images were also generated from the source images.

### Artificial intelligence-based 3-D reconstruction of intraabdominal organs and vessels around the stomach

Automated segmentation of the stomach, liver, GB, pancreas, spleen, rib, skin, and abdominal wall was performed on the portal phase CT image using a deep learning algorithm based on fine-tuned 3D U-Net. 3D U-Net is a specialized deep learning algorithm for biomedical image segmentation, and this algorithm used its own fine-tuned model with learning from radiologists-annotated clinical data. On the early arterial and the portal phase CT images, biomedical engineers used semi-automatic segmentation software (AVIEW, Coreline Soft, Seoul, Korea) and segmented upper abdominal vessels which were essentially needed for making a surgery-oriented 3-D model as follows: aorta, celiac artery, left and right gastric arteries, splenic artery, common hepatic artery, proper hepatic artery, left hepatic artery, right hepatic artery, aberrant hepatic artery if present, gastroduodenal artery, left and right gastroepiploic arteries, inferior vena cava, portal vein, splenic vein, left gastric vein, and left and right gastroepiploic veins. For 3-D reconstruction, 3-D masks of organs and vessels were obtained from this segmentation process and inspected by one radiologist with 19 years of experience in abdominal imaging.

### Modeling and texturing of 3-D models, including patient-specific pneumoperitoneum model generation

The following is the description of the modeling and texturing process after the 3-D reconstruction of intraabdominal organs and vessels around the stomach from the CT angiography. Mesh data of the intraabdominal organs and vessels were created using the 3-D reconstruction function of the Visualization Toolkit (VTK) library (https://vtk.org). The VTK is an open-source software for 3D rendering and a suite of widgets for 3D interaction. Simultaneously, patient-specific pneumoperitoneum model mesh data is generated using a deformation algorithm based on the patient’s demographic and clinical features.

Subsequently, the texture UV coordinates of the organs and vessels mesh data were created and the position was adjusted based on the artery and vein feature points using the Blender tool (https://blender.org). Blender is an open-source 3D computer graphics software toolset used for creating visual effects, art, interactive 3-D applications, and virtual reality. All the 3-D models were transferred to a client computer equipped with RUS™ software.

### Preoperative planning and simulation

The surgeon simulated patient-specific preoperative planning for port placement with instrumentation and virtual anatomy navigation using a client computer equipped with RUS™. Surgeons may also bring recorded preoperative planning and simulation to the operation room for intraoperative use. **Video 1** demonstrates an exemplary scene of virtual port placement and anatomy simulation according to the six pre-defined points of interest.

### Robotic gastrectomy for gastric cancer procedures with RUS™

All surgeries were performed using da Vinci Xi system (Intuitive, Sunnyvale, CA) by two surgeons (Kim YM and Hyung WJ) who had 8- and 16-year experience in gastric oncologic surgery, respectively. Robotic distal gastrectomy procedure was described in detail previously (2, 10). RUS™ client computer was connected to the robot console *via* HDMI port to integrate the RUS images using the TilePro™ program, which can display multiple digital sources (11, 12). During surgery, the surgeon frequently turned on the TilePro™ program for RUS™ images to navigate the correlating vessels and intraabdominal organs and align with the real-time surgical view.

With the patient supine, an 8-mm diameter trocar was inserted just below the umbilicus and the operating table was placed in a 15-degree reverse Trendelenburg position under 12 mmHg pneumoperitoneum. The surgeon could refer to the preoperative simulated images or videos of virtual trocar placement during the actual port placement. The dissection procedure corresponded with the six pre-defined points of interest. Using RUS™, the surgeon could identify details of vascular anatomy which are essential for lymphadenectomy. Intricacies of the virtual vascular anatomy were visualized at the console by RUS™ manipulation by the surgeon. The **Videos 2**-**7** offer representative operative scenes according to the six pre-defined points of interest.

### Outcome measures

The primary outcome was the feasibility of RUS™ for robotic gastrectomy. The feasibility was evaluated as the successful use of RUS™ without any error in delivering the 3-D model or inability to perform robotic gastrectomy by generating a 3-D model until its use for operation. Secondary outcomes were the turnaround time, the accuracy of detecting vascular anatomy with its variations, and the comparison of perioperative outcomes with a control group. The turnaround time was defined as the time from the patient’s CT DICOM file and demographic information transfer until the creation of a patient-specific 3-D model for RUS™ use. When a 3-D model is developed, the feasibility of regular operation is checked so that the anatomical structures can be reviewed before surgery. We assessed the 3-D model information regarding the origin, location, and variations of vessels encountered when performing robotic subtotal gastrectomy. We compared the accuracy of the anatomy of each blood vessel identified by RUS™ with the actual intraoperative findings. We measured the distance from each vascular structure to the specific reference point. Distance from the reference point was measured using the function of RUS™ and confirmed by measuring distance using a flexible ruler during the surgery.

### Statistical analysis

We calculated the propensity-scores and reported continuous variables with mean (± standard deviation) or median (± interquartile range) depending on whether the variables had normal distribution or not and used Student’s t-test or Mann-Whitney U test when comparing with the control group. We reported categorical variables with numbers (percentage) and performed Chi-square or Fisher’s exact test as appropriate. *P* value <0.05 was considered significant. All statistical analyses were performed using R packages (Version 4.2.0; R Foundation for Statistical Computing, Vienna, Austria).

## Results

### Patients characteristics

Between September 2021 and March 2022, we enrolled 36 patients using surgical navigation for robotic gastrectomy in this single-arm prospective study. Six patients were excluded due to two withdrawals, two required total gastrectomy which was confirmed by preoperative endoscopy, one refused robotic surgery, and one could not use Tilepro™ due to the mechanical problem related to Tilepro™ connection. Finally, 30 patients were included in the analysis. Of these, 13 (43.3%) were male, and the median age was 53.6 years. The characteristics of patients in this study are shown in **Table 1**. No patients had any comorbidities that would make robotic gastrectomy with lymph node dissection and preoperative CT with angiography unsafe. All examinations were performed and transferred to the surgeon’s console successfully. All 30 robotic gastrectomies were performed without any problems.

**Table 1:** Comparison between experimental (RUS™ applied) and control group after propensity score matching.

	Matched groups		
	No. (%)		
Characteristics	RUS applied (N = 30)	Non-RUS applied (N = 30)	*P* value
*Age, mean (SD), years	53.6 (10.8)	54.0 (10.8)	0.896
*Sex			>0.999
Male	13 (43.3)	12 (40.0)	
Female	17 (56.7)	18 (60.0)	
*BMI, mean (SD), kg/m2	23.2 (2.8)	23.1 (3.2)	0.892
ASA score			>0.999
1,2	24 (80.0)	23 (76.7)	
3,4	6 (20.0)	7 (23.3)	
Previous abdominal surgery			0.144
Yes	11 (36.7)	5 (16.7)	
No	19 (63.3)	25 (83.3)	
Clinical T stage			0.765
cT1	20 (66.7)	21 (70.0)	
cT2	6 (20.0)	3 (10.0)	
cT3	3 (10.0)	4 (13.3)	
cT4a	1 (3.3)	2 (6.7)	
Clinical N stage			0.506
cN0	27 (90.0)	25 (83.3)	
cN1	1 (3.3)	4 (13.3)	
cN2	2 (6.7)	1 (3.3)	
*Extent of lymph node dissection			0.784
<D2	21 (70.0)	19 (63.3)	
D2	9 (30.0)	11 (36.7)	
*Reconstruction			0.842
Billroth I	24 (80.0)	24 (80.0)	
Billroth II	3 (13.3)	4 (10.0)	
Roux-en-Y gastrojejunostomy	3 (6.7)	2 (10.0)	
Anesthesia time, mean (SD), min	218.6 (34.7)	230.3 (50.3)	0.299
Operation time, Mean (SD), min	177.1 (34.9)	193.9 (50.3)	0.137
Robot console time, Mean (SD), min	129.3 (34.4)	147.4 (48.3)	0.101
Estimated blood loss, Mean (SD), ml	46.5 (37.7)	44.0 (43.1)	0.809
Tumor size, Mean (SD), mm	29.4 (18.9)	29.2 (18.7)	0.958
Pathologic T stage			0.849
pT1	22 (73.4)	20 (66.7)	
pT2	3 (10.0)	3 (10.0)	
pT3	4 (13.3)	4 (13.3)	
pT4a	1 (3.3)	3 (10.0)	
Pathologic N stage			0.814
pN0	22 (73.3)	20 (66.7)	
pN1	4 (13.3)	5 (16.7)	
pN2	2 (6.7)	4 (13.3)	
pN3	2 (6.7)	1 (3.3)	
AJCC 8^th^			0.718
Stage I	23 (76.7)	20 (66.6)	
Stage II	4 (13.3)	5 (16.7)	
Stage III	3 (10.0)	5 (16.7)	

SD, standard deviation; BMI, body mass index; ASA, American Society of Anesthesiology; T stage, Tumor stage; N stage, Lymph Nodes stage; AJCC, American Joint Committee on Cancer.

*Matched variable: Age, Sex, BMI, Extent of lymph node dissection, Reconstruction.

### Feasibility

The application of patient-specific surgical navigation using RUS™ for robotic gastrectomy for gastric cancer was successful in all 30 patients. The whole process of patients-specific surgical navigation was carried out without any transfer problem or system error. No intraoperative events related to the application of RUS™ occurred. There were no intraoperative unintended vessel injuries requiring combined resection or operation on other organs. There were five postoperative complications which were not related to RUS™ use. They included intraabdominal abscess, pneumonia, urinary tract infection, profuse drain fluid with hypoalbuminemia requiring diuretics use, and unknown C-reactive protein elevation and were managed conservatively.

The turnaround time was less than 72 hours in all patients. The turnaround time was within 24 hours in 19 (63.3%) patients, within 36 hours in 9 (30.0%), and within 48 hours in 2 (6.7%) patients. Thus, it took less than a day in 63.3% and less than two days in 93.3% of patients.

### Accuracy of vascular anatomy identification

In all 30 patients, all vascular anatomy around the stomach was accurately reconstructed and precisely identified, completed, and matched with operative findings (**Table 2**). The omental branches of left gastroepiploic vessels were preserved in all 30 patients, which resulted in no remnant omental infarction after partial omentectomy. The right gastroepiploic vein drained to the gastrocolic trunk in 22 (73.3%) patients, while the other 6 (26.7%) drained to the superior mesenteric vein directly without forming the gastrocolic trunk. The left gastroepiploic vein was successfully ligated without damage to the accessory right colic vein. In 16 (53.3%) patients, right gastroepiploic artery branched off the infrapyloric artery, and in 14 (46.7%) patients, the gastroduodenal artery branched off the infrapyloric artery. In the latter case, the infrapyloric artery was ligated separately without any injury.

**Table 2:** Anatomic information of vessels of interest during robotic subtotal gastrectomy.

Patient no.	RGEV drains into	IPA from	RGA from	^1^RGA branch distance (mm)	^†^LGV drains into	^2^LGV branch distance (mm)	^3^LGA branch distance (mm)	Aberrant left hepatic artery
1	GCT	GDA	GDA	4.7	PV(p)	28.5	3.2	Absent
2	GCT	GDA	PHA	5.3	SV(a)	23.4	10.4	Absent
3	SMV	RGEA	PHA	4.8	PV(p)	17.5	11.5	Absent
4	GCT	GDA	PHA	6.9	PV(p)	20.2	13.7	Accessory
5	SMV	RGEA	GDA	6.8	Left portal vein	–	3.6	Absent
6	GCT	RGEA	GDA	3.8	PV(p)	15.7	6.4	Absent
7	SMV	RGEA	LHA	2.5	SV(a)	16.8	5.1	Absent
8	GCT	RGEA	PHA	2.7	PV(p)	23.0	5.1	Absent
9	GCT	GDA	PHA	9.1	PV(p)	11.1	7.6	Absent
10	SMV	RGEA	^*^Trifurcation	–	PV(p)	21.5	3.4	Absent
11	GCT	GDA	PHA	14.1	SV(a)	30.0	7.6	Absent
12	GCT	RGEA	PHA	8.6	PV(p)	19.4	3.6	Replaced
13	GCT	GDA	PHA	7.1	PV(p)	25.0	9.0	Absent
14	GCT	RGEA	PHA	4.5	SV(a)	19.3	^*^Trifurcation	Absent
15	GCT	GDA	LHA	9.0	PV(p)	21.1	^*^Trifurcation	Absent
16	SMV	RGEA	PHA	2.5	PV(p)	22.2	4.1	Accessory
17	GCT	GDA	PHA	9.3	PV(p)	24.0	6.1	Absent
18	GCT	RGEA	PHA	6.4	PV(p)	19.3	3.6	Absent
19	GCT	GDA	PHA	10.6	SV(a)	17.8	7.0	Absent
20	GCT	GDA	LHA	12.9	SV(a)	22.2	9.0	Absent
21	SMV	RGEA	PHA	5.2	SV(a)	20.0	3.5	Absent
22	GCT	GDA	PHA	6.1	PV(p)	23.0	5.6	Absent
23	SMV	RGEA	PHA	7.8	SV(a)	29.4	^*^Trifurcation	Absent
24	GCT	RGEA	GDA	3.2	PV(p)	19.9	5.4	Accessory
25	GCT	GDA	GDA	4.8	SV(a)	18.7	^*^Trifurcation	Absent
26	GCT	RGEA	LHA	6.9	SV(a)	33.0	12.2	Replaced
27	GCT	RGEA	^*^Trifurcation	–	SV(a)	35.4	^*^Trifurcation	Absent
28	GCT	GDA	GDA	4.0	PV(a)	16.8	5.6	Absent
29	SMV	GDA	PHA	5.0	SV(a)	25.1	4.6	Absent
30	GCT	RGEA	PHA	11.4	PV(p)	25.2	5.7	Absent

RGEV, right gastroepiploic vein; GCT, gastrocolic trunk; SMV, superior mesenteric vein; IPA, infrapyloric artery; GDA, gastroduodenal artery; RGA, right gastric artery; PHA, proper hepatic artery; LHA, left hepatic artery; LGV, left gastric vein; PV, portal vein; SV, splenic vein; LGA, left gastric artery.

^1^ RGA branch distance is measured from the RGA root to the point the common hepatic artery divides into the GDA and the PHA.

^2^LGV branch distance is measured from the LGV drainage point to the point the common hepatic artery divides into the GDA and the PHA.

^3^LGA branch distance is measured from the LGA root to the point the celiac trunk divides into the common hepatic artery and the splenic artery.

^*^Three or more vessels are branched at the same point.

^†^A or P in parentheses indicates the positional relationship with the common hepatic artery and the LGV drainage point; a, anterior; p, posterior.

Right gastric artery originated from the proper hepatic artery in 18 (60.0%) patients, from the gastroduodenal artery in 6 (26.7%) patients, from the left hepatic artery in 4 (26.7%) patients, and trifurcation at the branching point of the proper hepatic and the gastroduodenal artery in 2 (6.7%) patients. The mean distance from the branching point of the proper hepatic and gastroduodenal artery to the right gastric artery root was 5.4mm (range:0–14.1mm). The left gastric vein drained into the main portal trunk in 17 (56.7%) patients (15 posterior and two anterior to the common hepatic artery) and into the splenic vein anterior to the common hepatic artery in 12 (40.0%), and into the left portal vein in 1 (3.3%). The mean distance from the branching point of the common hepatic and splenic artery to the left gastric vein was 21.5 mm (range: 2.2–35.4 mm). Left gastric artery was branched as a trifurcation of the celiac trunk in 5 (16.7%) patients. It was bifurcated from the common trunk of the common hepatic and splenic artery in 25 (83.3%) patients. The mean distance from the bifurcation of the common hepatic and splenic artery to the origin of the left gastric artery was 6.5 mm (range: 0–13.7 mm). All vascular variations of the aberrant left hepatic artery were identified. There were 5 (16.7%) patients who had the aberrant left hepatic artery branching from the left gastric artery; from an accessory left hepatic artery in three patients, and from a replaced left hepatic artery in two patients. Additionally, all gastric branches from the aberrant left hepatic artery were identified. The aberrant left hepatic artery was preserved in all five patients. Information regarding these arterial variations enabled surgeons to avoid accidental hemorrhage and ischemic liver damage during surgery (**Video 8**).

### Operative data and short-term outcomes compared with the control group

**Figure 3:**
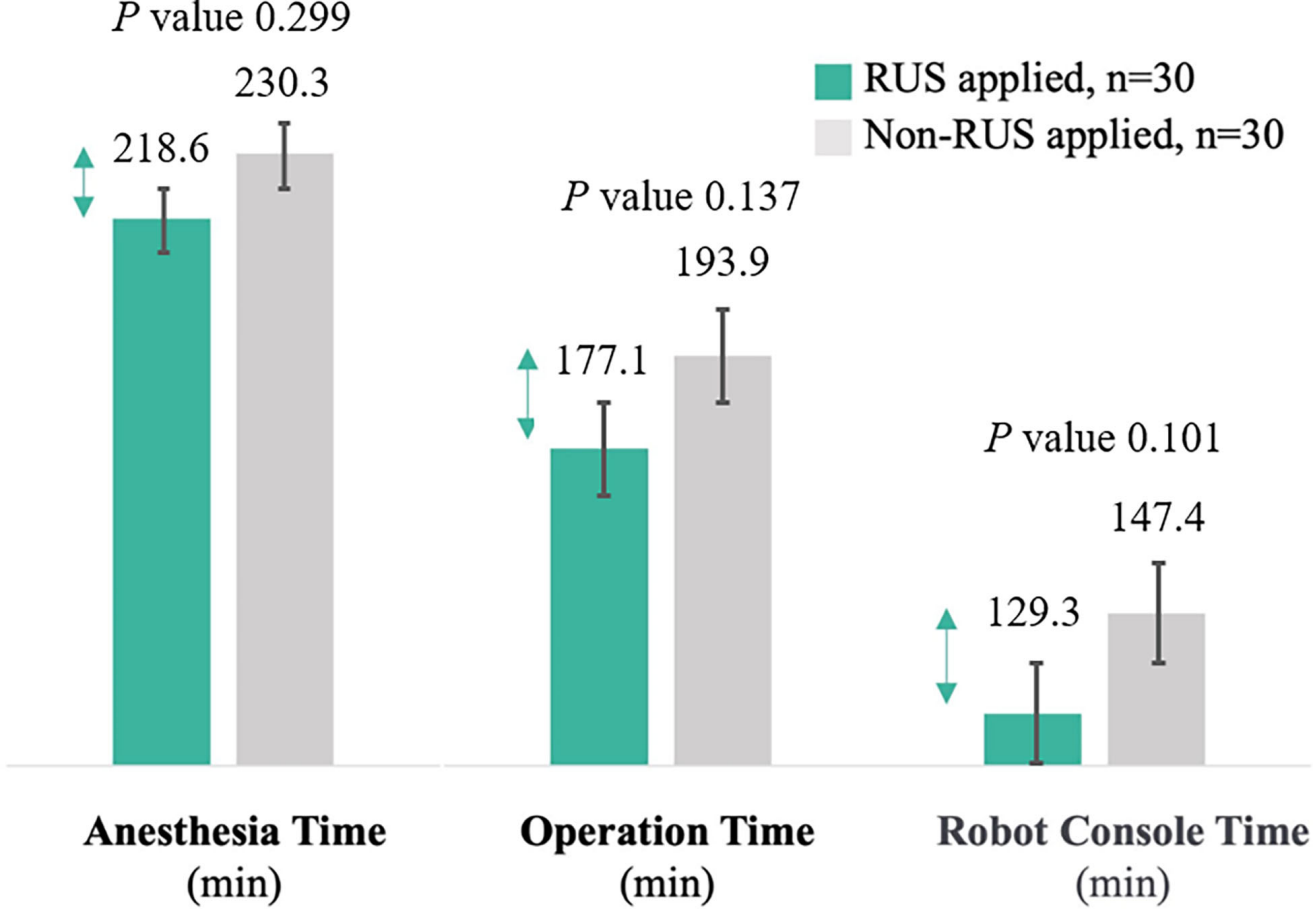
Comparison of anesthesia, operation, and console time between the experimental and control group (Error bars are used on graphs to indicate the standard error).

After propensity matching, the experimental and control groups were well-balanced in demographics and operative features. The operative data and short-term outcomes between the experimental and control group were comparable. The number of harvested lymph nodes and estimated blood loss were similar between the two groups (**Table 1**). The experimental group showed shorter anesthesia time (218.6 min *vs*. 230.3 min; *P*=0.299), operative time (177.1 min *vs*. 193.9 min; *P*=0.137), and console time (129.3 min *vs*. 147.4 min; *P*=0.101) than the control group, although the differences were not statistically significant. The proportions of the reduction in anesthesia time, operative time, and console time were 5.1%, 8.7%, and 12.3%, respectively (**Figure 3**).

## Discussion

In this prospective observational study, patient-specific 3-D surgical navigation system for robotic gastrectomy for gastric cancer is clinically feasible and a maximum turnaround time of 3 days is acceptable. This surgical navigation system completely visualized all the vascular anatomy required for gastrectomy in 3-D models, including all vascular variations and all information regarding the position of the vessels, without any error during preoperative planning and intraoperative navigation.

There have been various attempts to provide virtual anatomical information during minimally invasive surgery (13, 14). However, most of them offered only the reconstructed images of anatomical structures without virtual operative environment mimicking actual operation. Reconstruction processes are time-consuming, laborious, and difficult. Moreover, deformation occurs due to the difference in the position of the patient during CT scan, and the registration of artery and vein images obtained at different times are not widely available with technical challenges (15).

This new patient-specific 3-D surgical navigation system, RUS™, has key features that make virtual images like the actual operation findings. The patient-specific pneumoperitoneum model enables preoperative planning such as trocar placement and can postulate a virtual view as real operative view by inserting a virtual 30° down view camera through a virtual camera trocar. The effect of the deformation of organs and vessels caused by gravity due to the differences in the position of the patient during CT scan and surgery was minimized using a CT scan in a 15-degree reverse Trendelenburg position, the same position as during gastrectomy. Pre-defined points of interest make it easy to manipulate the virtual model during surgery. An algorithm for artery and vein image registration facilitated accurate identification of the relative position between arteries and veins resembling actual operation.

The anatomical diversity of vessels around the stomach, as if all patients in this study were different, is a major challenge for surgeons when performing gastric cancer surgery since skeletonizing and managing vessels are crucial for thorough lymphadenectomy (16–18). Moreover, there were frequent vascular variations. The navigation system provided patient-specific information on the relevant vascular information and led the surgeon to branching sites and facilitated lymphadenectomy around the vessels. The surgeon can eliminate the process of careful dissection to find the small or deep-seated vessels based on guesswork or knowledge from previous experience without fear of vascular injury. In this study, there were no accidental bleeding or damage to other organs during surgery. These advantages of a surgical navigation system have the potential to reduce operation time and console time.

The patient-specific 3-D surgical navigation system provides a fair environment by delivering accurate anatomical information on patients operated by removing the anatomical knowledge barrier between novice and experienced surgeons. It makes it easier and safer for surgeons, especially inexperienced surgeons, to perform complex minimally invasive surgery. Furthermore, this can lead to patient-specific surgery not based on anatomy case studies or textbook-based knowledge but on individual anatomy. Thus, this patient-specific 3-D surgical navigation system offers a comprehensive platform for truly helping surgeons from preoperative planning to intraoperative surgical navigation.

This study has some limitations. First, although we used a control group to compare with the experimental group, this is not a randomized controlled study. Second, we included only 30 patients to verify the feasibility. The small number of study patients hindered the identification of the clinical benefits of this navigation system compared with the control group. Third, we applied this navigation system only for robotic gastrectomy, which has a multi-display function. Thus, applying this system during laparoscopic surgery, which requires an additional 3-D monitor during surgery and intraoperative manipulation of the system, is rather limited compared with robotic surgery because surgeons are in an entirely aseptic condition, unlike the robotic console surgeon.

In addition, the system requires improvement. An automated port placement recommendation function would help inexperienced surgeons easily decide the trocar position. Automatic tumor visualization to determine the resection extent with adequate resection margins would help surgeons eliminate additional tumor localization processes during surgery. Automatic synchronization of the virtual camera movement to follow the actual operative camera position would make surgeons free from intraoperative controlling of the system and improves intraoperative control of surgical navigation during surgery, especially for laparoscopic surgery.

To the best of our knowledge, this is the first study to prove the feasibility and applicability of a patient-specific virtual 3-D navigation system for robotic gastrectomy for gastric cancer with an acceptable maximum turnaround time of 2 days. Surgeons were able to perform patient-specific preoperative planning and intraoperative navigation with complete anatomical information required for gastrectomy in 3-D models without any error. This study showed the potential for the patient-specific virtual 3-D navigation system to facilitate robotic gastric cancer surgery in clinical practice. Larger sample size randomized studies are needed to further assess the development and clinical efficacy of this patient-specific 3-D surgical navigation system in various operative environments, to provide more robust evidence for the routine application.

## Conclusion

Patient-specific 3-D surgical navigation system for robotic gastrectomy for gastric cancer patients is feasible and applicable with an acceptable turnaround time. This surgical navigation system enables patient-specific preoperative planning and intraoperative navigation by visualizing all the anatomy required for gastrectomy in 3-D models.

## Data availability statement

The raw data supporting the conclusions of this article will be made available by the authors, without undue reservation.

## Ethics statement

The studies involving human participants were reviewed and approved by the Institutional Review Board (IRB) of Severance Hospital, Yonsei University Health System (1–2021–0036). The patients/participants provided their written informed consent to participate in this study.

## Author contributions

WH and YK had full access to all the data in the study and takes responsibility for the integrity of the data and the accuracy of the data analysis. Study concept and design: YK and WH. Acquisition of data: SP and KK. Analysis and interpretation of data: SP, YK and WH. Drafting of the manuscript: SP, YK and WH. Critical revision of the manuscript for important intellectual content: KK and WH. Statistical analysis: SP and YK. Administrative, technical, or material support: YK and WH. Obtaining funding: YK and WH. Study supervision: YK and WH. All authors contributed to the article and approved the submitted version.

## Supplementary material

Supplementary Video 1 A patient-specific 3-D surgical navigation for preoperative planning for port placement with instrumentation and virtual anatomy navigation according to the six pre-defined points of interest.

Supplementary Video 2 Dissection of left gastroepiploic vessels area.

Supplementary Video 3 Dissection of right gastroepiploic vein area.

Supplementary Video 4 Dissection of right gastroepiploic artery and infrapyloric artery area.

Supplementary Video 5 Dissection of right gastric artery area.

Supplementary Video 6 Dissection of left gastric vein area.

Supplementary Video 7 Dissection of left gastric artery area.

Supplementary Video 8 Dissection in case with left gastric artery variation with preservation of replacement left hepatic artery.
